# A Flexible Pressure Sensor with Ink Printed Porous Graphene for Continuous Cardiovascular Status Monitoring

**DOI:** 10.3390/s21020485

**Published:** 2021-01-12

**Authors:** Yuxin Peng, Jingzhi Zhou, Xian Song, Kai Pang, Akram Samy, Zengming Hao, Jian Wang

**Affiliations:** 1 Department of Sports Science, Zhejiang University, Hangzhou 310058, China; yxpeng@zju.edu.cn (Y.P.); jzz@zju.edu.cn (J.Z.); zengminghao@zju.edu.cn (Z.H.); pclabeeg@zju.edu.cn (J.W.); 2 Department of Polymer Science and Engineering, Zhejiang University, Hangzhou 310027, China; pangkai2015@163.com; 3 Department of Civil Engineering and Architecture, Zhejiang University, Hangzhou 310058, China; akram@zju.edu.cn

**Keywords:** flexible pressure sensor, porous graphene, shear force elimination, blood pressure estimation

## Abstract

Flexible electronics with continuous monitoring ability a extensively preferred in various medical applications. In this work, a flexible pressure sensor based on porous graphene (PG) is proposed for continuous cardiovascular status monitoring. The whole sensor is fabricated in situ by ink printing technology, which grants it the potential for large-scale manufacture. Moreover, to enhance its long-term usage ability, a polyethylene terephthalate/polyethylene vinylacetate (PET/EVA)-laminated film is employed to protect the sensor from unexpected shear forces on the skin surface. The sensor exhibits great sensitivity (53.99/MPa), high resolution (less than 0.3 kPa), wide detecting range (0.3 kPa to 1 MPa), desirable robustness, and excellent repeatability (1000 cycles). With the assistance of the proposed pressure sensor, vital cardiovascular conditions can be accurately monitored, including heart rate, respiration rate, pulse wave velocity, and blood pressure. Compared to other sensors based on self-supporting 2D materials, this sensor can endure more complex environments and has enormous application potential for the medical community.

## 1. Introduction

Pulse signals are generated by the periodic contraction and relaxation of the heart muscle, and it is deeply affected by cardiovascular status. Thus, pulse signals contain various pieces of physiological information and have already been widely adopted in Western clinical examinations [1] and traditional Chinese medicine [2]. Monitoring detailed pulse information in real time can significantly reduce accidents due to cardiovascular problems in individuals [3]. However, due to the high cost and large scale of the pulse monitoring device, only the cardiovascular status of patients with severe conditions can be monitored in real time [4]. Thus, establishing a low-cost and wearable pulse monitoring system has great significance in precision medicine, health engineering, and chronic disease rehabilitation.

In the last few years, many sensors have been developed for the precise monitoring of pulse signals. From the working principle perspective, state-of-the-art pulse acquisition technologies can be divided into the photoelectric pulse sensor [5], ultrasonic pulse sensor [6], and pressure pulse sensor [7]. The photoelectric pulse sensor is prone to conduct large-scale manufacture, and the ultrasonic pulse sensor has high robustness. However, these two kinds of pulse sensor cannot collect vital information for pulse taking, such as vessel elasticity [8], which directly affects the transmural pressure of blood vessels. In this aspect, the principle of the pressure pulse sensor is identical to the pulse sensation of physicians, which makes it a better choice for digitalizing the pulse-taking procedure. Since the pulse is a weak human motion, the pressure sensor requires surface compatibility and sensitivity. From this perspective, flexible and stretchable pressure sensors with extraordinary biocompatibility and high sensitivity are preferable for precise pulse measurement [9,10]. 

Various flexible and stretchable sensors were recently proposed for pressure sensing, including organic field-effect transistors (OFETs) [11], liquid metal [12], conductive polymers [13,14], and low dimensional materials [15,16]. Due to high sensitivity and small volume, low dimensional materials are widely investigated in the flexible electronics community, such as metal nanomaterials, carbon nanotubes, and graphene. Metal nanomaterials such as AgNPs, AgNWs, and AuNWs process high sensitivity, yet their high costs prevent the sensor from having wide-range application [17,18,19,20,21]. Carbon nanotubes (CNT) such as single-wall CNT (SWCNT) and multi-wall CNT (MWCNT) are cheap, but their electromechanical properties are hard to control, which is still a dilemma in large-scale manufacturing [22,23]. On the other hand, the raw material cost of graphene is low, and its performance is stable for the manufacturing procedure, which makes it a desirable material for flexible electronics design. 

As a three-dimensional graphene macroscopic assembly, porous graphene (PG) possesses excellent electromechanical properties when encountering external pressure stimuli [24]. With deliberately organized preparation conditions, this material can reach an extremely high sensitivity and accurately record small signals such as pulse waveform shape. However, high-temperature forming is required by high-performance PG manufacture, where flexible substrates cannot withstand such a severe manufacturing environment [25]. Subject to this specific foaming procedure, almost all studies on PG-based sensors were fabricated ex situ, which makes automatic manufacture hard to conduct [26]. Thus, in situ forming for PG is required in real production circumstances.

Moreover, there is another major problem preventing this kind of self-supporting graphene material from being widely applied: They cannot withstand shear forces. This drawback severely reduces its robustness when mounting on the complex skin surface [27]. Conventional manufacturing procedures usually employ silicon rubber as sealing material to protect functional elements, which, in this case, worsens the shear force situation of the PG due to its viscoelastic property [28,29]. Thus, a novel sealing approach should be developed to eliminate the effect of the shear force and excavate the potential of ultra-sensitive PG.

In this paper, we propose a novel pressure sensor conducted by ink-printed PG. This material is manufactured by ink printing and in situ foaming, which has the potential for large-scale automatic manufacturing. The PG is protected by plastic encapsulating to eliminate most of the shear force, and it is sealed by silicon rubber to gain skin compatibility. With accurate patterning and proper reducing procedures, this sensor exhibits extraordinary sensitivity (53.99/MPa) and high resolution (<0.3 kPa) even under a tiny external stimulus (5 μm compression). Both the robustness and stability of the sensor are desired so that it can then be attached to different areas of the human body to evaluate cardiovascular status, including breath frequency, heart rate, pulse velocity, and blood pressure. Results show that the sensor can be comfortably mounted on human skin, and the sensor output has a satisfying quality, which demonstrates its value and significance in cardiovascular status estimation and other medical applications.

## 2. Methodology

### 2.1. Materials

In this study, the graphene oxide (GO) suspension (16 mg/mL, 50–80 μm lateral width) was purchased from Hangzhou Gaoxi Technology Co., Ltd. (Hangzhou, China) for porous graphene preparation. Moreover, the laminated film was obtained from Yalan paper industry Co., Ltd (Guangzhou, China). with 50 μm thickness and the Ecoflex (Smooth-On 00-30) was purchased from BASF SE.

### 2.2. Fabrication Procedure

**Figure 1:**
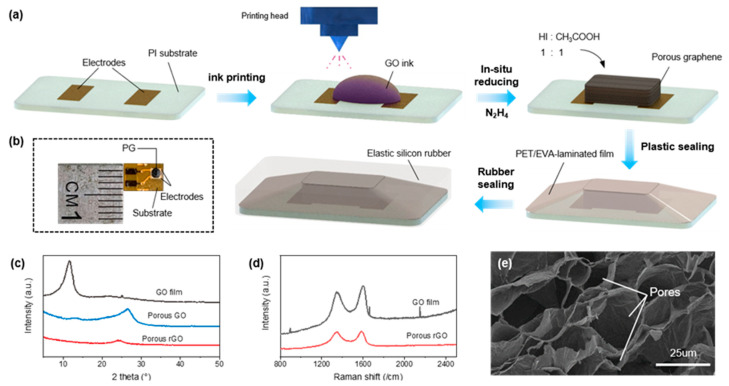
(**a**) Fabrication procedure of the proposed sensor, (**b**) photograph of the sensor, (**c**) XRD characterization of the porous graphene, (**d**) Raman spectrum of the porous graphene, and (**e**) SEM images of the porous graphene.

The fabrication process of the proposed pressure sensor is shown in Figure 1a. Electrodes were conducted on a polyimide (PI) substrate by electroless nickel and immersion gold (ENIG). The GO ink was then dispensed onto the ENIG manufactured electrodes by ink printing and the foaming reagents of 30% (*wt/wt*) N_2_H_4_ aqueous was sprayed on the dried GO film to construct the pressure-sensitive porous graphene (PG).

In order to improve the conductivity of the PG, the oxygen-containing groups of the GO were further removed by HI and CH_3_COOH (1:1) and dried for 2 h at 95 °C. All the printing and reducing procedures were conducted in an in situ manner. A PET/EVA-laminated film was adopted to seal the PG by hot pressing it upon the substrate edge. In the end, the whole device was encapsulated via silicon rubber (Ecoflex) to obtain skin compatibility. The photograph of the proposed sensor is shown in Figure 1b.

### 2.3. Characterization

The X-ray diffraction (XRD) of the sensor was investigated on an X’ Pert Pro (Malvern PANalytical, Netherlands) diffractometer using monochromatic Cu 17 Kα1 radiation (λ = 1.5406 Å) at 40 kV. The measurement for Raman spectra was conducted through a Via-Reflex Raman microscopy (Renishaw, Gloucestershire, UK) with an excitation wavelength of 532 nm. Moreover, an electromechanical universal testing platform (Instron Legend 2344, Norwood, MA, USA) was employed to evaluate the electromechanical performance of the proposed sensor, and the measurement for the resistance variation was conducted by Keithley 2400 Source Meter.

## 3. Experiments

### 3.1. Microstructure and Morphology

Figure 1c shows the XRD image of the as-prepared PG. In the original status, the characteristic peak (001) of the dried GO film appeared at 11.6°. After the in situ foaming of the aqueous N_2_H_4_, the GO had a slight restoration, displaying the significantly declining (001) peak and the appearance of the (002) peak at 26°. By further reducing with HI/CH_3_COOH, the disappearance of the GO peak indicates that almost all oxygen-containing groups were successfully removed. The Raman spectrum is shown in Figure 1d, where strong D bands and G bands can be clearly observed at approximately 1350 cm^−1^ and 1580 cm^−1^, respectively. The D/G band of the porous reduced GO (rGO) was higher than the GO film, which indicates that the number of sp2 domains increased in the 3D structure. These results confirm the successful reduction of GO in another term.

**Figure 2:**
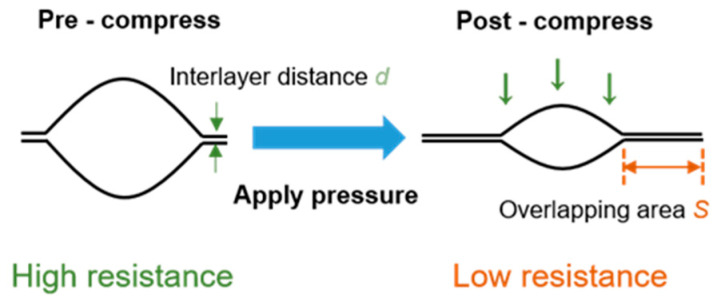
Sensing principle of the PG.

Figure 1e shows the SEM image of the PG with 1000 times magnification, where a vast number of pores can be observed in the PG. Due to the change in resistance of the graphene conductive path within the material during deformation, the porosity of PG generated its sensing ability. As shown in Figure 2, when enduring external pressure, the overlapping area of the compressed pore increased. The PG resistance consequently decreased following the equation of contact resistance:

(1) $$R_{contact}=k\times \frac{d}{S}$$

where *k* is the contact resistivity, *d* represents the interlayer spacing, and *S* indicates the overlapping area volume.

### 3.2. Pressure Sensing Property

**Figure 3:**
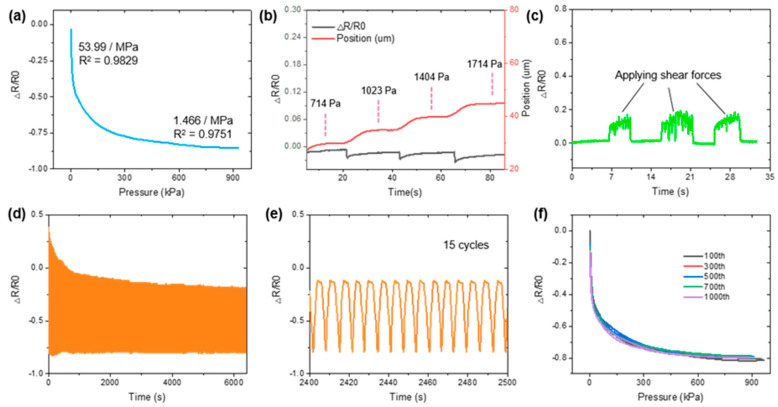
Electromechanical and sensing performance of the proposed sensor: (**a**) sensor output calibration, (**b**) resolution performance, (**c**) sensor output when enduring unexpected shear forces, (**d**) the 1000-cycle repeatability test, (**e**) detailed view of the repeatability test, and (**f**) hysteresis analysis for different loops.

The mechanical performance of the as-prepared sensor is presented in this paper. Figure 3a shows that the maximum gauge factor (GF) reached 53.99/MPa during the first stage (less than 0.1 MPa). Since the interlayer spacing *d* is small, in this stage, most of the resistance changes are caused by the rapid variation of overlapping area *S*. The available deformation volume of the pores is reduced with the increasing compression level. Thus, the increment of overlapping area *S* encounters its limit after a certain pressure value, and the change of interlayer spacing *d* dominates the resistance variation. Following the contact resistance law, the GF decreases significantly. In this case, from Figure 3a, this change happened at approximately 0.3 MPa, and the GF decreased towards 1.466/MPa. To sum up, the pore deformation provides the sensor high sensitivity for small pressure detection, and the interlayer spacing grants the sensor the ability of large pressure monitoring. Four 5 μm-compression steps were conducted to investigate the PG resolution with approximately 0.3 kPa pressure, where stair shapes can be observed in Figure 3b. From the above results, the sensing range of the sensor is approximately 0.3 kPa–1 MPa.

In practice, nanomaterial-based sensors are vulnerable due to the surface complexity of human skin. The intensive shear force generated by frequent friction usually causes the failure of sensing materials. In order to deal with this problem, this paper provides an extra plastic sealing method for shear force elimination. To test the protection effect, friction was applied by rubbing the sensor dozens of times during the experiment, and its baseline remained intact, as shown in Figure 3c. This result demonstrates the sensor’s robustness when encountering unexpected shear forces. In the end, for repeatability, the sensor exhibited the desired performance during a 1000-cycle test with stable hysteresis loops, as shown in Figure 3d–f.

Notice that the curve peak of the PG decreased by approximately 30% in Figure 3d, and the reason for it can be explained as follows. Since only the edge of the substrate was sealed by the PET/EVA film, air remained in the encapsulation structure before it was first pressed, which was expelled after continuously pressing. In this case, the imbalanced air pressure made the sensor shrink a little bit. Consequently, the thinner structure reduced the available deformation range of the PG and scaled down the peak value of the following curves. After about 100 cycles, most of the air was expelled, and the sensor performance was stable, as shown in Figure 1f. If a product level device is required, the manufacturer can expel the air before delivery. In this way, the sensor can work in the stable range at the client terminal. Although the pressure difference between the internal and external environment may increase the stress at the sealing edge, the air can be expelled from the wall of PET film due to its moderate air permeability [30]. Thus, instead of breaking the sealing edge, the sensor is not prone to crashing after enduring continuous pressing, as suggested by the results in Figure 3d,e.

Some state-of-the-art pressure sensors based on graphene are compared with this work in Table 1. It can be seen that, although some materials have high GFs, such as triode-mimicking graphene and graphene nanoplatelet foams, their resolution is not desired to capture small signals [16,31]. On the other hand, despite the graphene oxide sponge having a high resolution, both its GF and detection range are low, which limits its application range compare to the proposed sensor [32]. Additionally, no friction test was conducted for the abovementioned sensors, and therefore their feasibility for complex surface environments could not be confirmed.

### 3.3. Protection Principle

**Figure 4:**
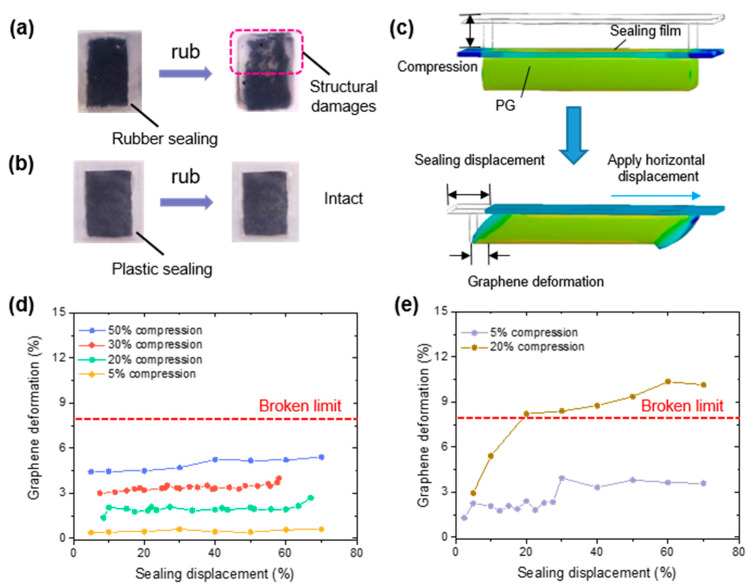
Protection principles: (**a**) fragile PG with rubber sealing, (**b**) well-protected PG with plastic sealing, (**c**) 3D illustration of applying shear force in ANSYS, and sealing displacement versus graphene deformation curve of (**d**) a plastic-sealed sensor and (**e**) a rubber-sealed sensor.

Since silicon rubber is a viscoelastic body, it attracts the surface of PG and enormously increases the friction factor. In this case, all of the shear force is transmitted towards the PG surface, and the PG structure is prone to being destroyed, as shown in Figure 4a. On the other hand, the friction between the sensor and the skin surface directly causes deformations of the plastic sealing film. Since the plastic film’s surface roughness is relatively small, the friction factor of the interface between the PG surface and the plastic film can be controlled at a low level. Thus, only a little shear force is transmitted to the PG surface, preventing the PG from being damaged by the friction, as shown in Figure 4b.

ANSYS (ANSYS Inc, Canonsburg, PA, USA), an engineering simulation software, is adopted to conduct finite element analysis for theoretical and numerical solutions of the protection principle. The model was simulated in ANSYS by considering the normal contact and the tangential friction, and the properties are listed in Table 2.

The 3D illustration for shear force-caused deformation is shown in Figure 4c. For the proposed PG, approximately 8% horizontal deformation caused irreversible damages. In this case, if the PG deformation exceeded 8%, the sensor was considered to be destroyed. Figure 4d,e shows the sealing displacement versus graphene deformation curves with different compression circumstances. It can be seen that even with 50% vertical compression, the sensor structure remained functional under a 70% sealing film displacement. Considering the sensor height, the most severe circumstance was 90% vertical compression with 20% horizontal movement. Under this condition, the sensor displacement in the finite element analysis reached 8.39%, which is only, if any, slightly damaged. The result in Figure 3c supports the finite element analysis. However, for rubber sealing, the deformation of PG surface exceeded 8% with a 20% compression under 20% sealing film displacement, which suggests that this sealing method cannot work properly on human skin. These results correspond to the rubbing experiments in Figure 4a,b.

## 4. Cardiovascular Status Monitoring

### 4.1. System Establishment

**Figure 5:**
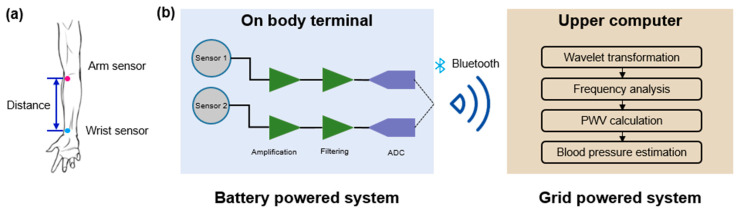
System architecture: (**a**) sensor positions on the human arm, where sensor 1 is the arm sensor and sensor 2 is the wrist sensor, and (**b**) system logic of the on-body terminal and the upper computer.

In this paper, the proposed sensors were mounted on both the forearm root and wrist to evaluate cardiovascular status, as shown in Figure 5a. The whole system for pulse measurement is shown in Figure 5b. The installation sites at the arm and wrist were above the cubital fossa vein and radius vein, which have obvious pulse signals and their distance does not change during human movement. Both sensors were attached to human skin by polyurethane (PU) film, which applied a little pre-pressure so that the pulse signal could be captured entirely.

Since the proposed sensors only generated shallow output sine pulse signals, they could be easily affected by the external environment, such as white noises and unexpected electromagnetic fields. Since pulse signals consist of low-frequency components, we established lowpass filters to eliminate frequency components beyond 25 Hz and amplified them subsequently. After being collected by an analog-digital converter (ADC) chip with a 1000 Hz sampling rate, the pulse signals were delivered to the upper computer by a Bluetooth transmitter. This whole on-body terminal was battery powered, making it able to be utilized in arbitrary environments.

The pulse data was then processed in the upper computer by Matlab 2020a. Respiration rate (RR) is a vital sign of cardiovascular status. Since breathing causes skin contraction and release, it generates a baseline for the pulse signal. Thus, we adopted a wavelet transformation with a sym10 basis to decompose the signal 10 times in the first place. The reconstruction signal of the 10th layer was considered the baseline (lower than 0.45 Hz).

Moreover, the heart rate (HR) was also evaluated by extracting pulse peaks. By seeking the local maximum within a 0.4 s interval, peaks were extracted by considering the maximum heart rate as 150 beats/min. This approach ensured that small waveform distortion would not affect the peak extraction [35]. Then, the frequency spectrum was drawn by fast Fourier transformation, which can be used in analyzing vessel health or cardio conditions [36,37].

Another essential indicator of cardiovascular status is blood pressure (BP). Individuals with cardiovascular diseases have risks of BP soaring during daily activity, leading to specific injuries or even death. For these cases, real-time BP monitoring is required. However, the traditional method requires an air cuff and a stethoscope, which cannot evaluate BP fluctuation in real time. In order to solve this problem, in this study, we employed a dual-sensor system to evaluate BP by calculating pulse wave velocity (PWV).

PWV is affected by vessel radius and elasticity, which can be represented by the Moens–Korteweg equation [38]:

(2) $$v=\sqrt{\frac{gEa}{\rho d}}$$

where *v* is the PWV, *g* is the gravitational acceleration, *E* is the vessel wall’s elastic modulus, *ρ* is the blood density, *a* is the vessel wall thickness, and *d* is the vessel radius.

Since the elastic module *E* has an exponential relationship with BP, the above equation can be used to estimate BP. Assuming that the vessel property is a constant for an individual within a short period, the relationship between PWV and BP can be represented as:

(3) BP=k⋅v+b

where *k* and *b* are undetermined parameters that depend on different body status. Calibrations can obtain these parameters in Equation (3) before the user conducts target activities, which guarantees that the vessel and blood status remain unchanged. The evaluation algorithm in this paper is the least square estimation.

### 4.2. Monitoring Experiments and Results

A volunteer from the Sports Department of Zhejiang University was chosen to wear the proposed sensory system. The volunteer was a healthy college student without a drug-use history. Since this experiment did not involve any invasive measurement or physiological stimulus, no ethical review was required.

**Figure 6:**
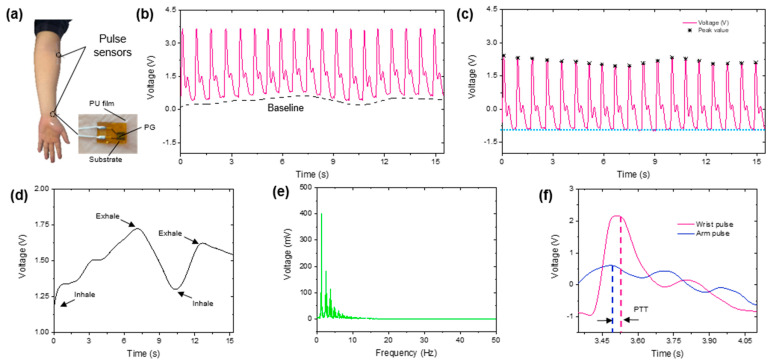
Signal processing result: (**a**) photograph of the sensor position, (**b**) original pulse signal received by the upper computer, (**c**) baseline elimination and pulse extraction, (**d**) respiration rate, (**e**) frequency spectrum, and (**f**) pulse transition time (PTT) calculation.

Sitting beside a desk, the volunteer raised his arm to three different heights to change the BP without altering vessel status. Each height was measured twice. Two sensors measured pulse waves on both the forearm root and the wrist, and the PWV was calculated by the distance and pulse transition time. Following commonly used methods [39], the sensor was covered by the PU film and then straightforwardly adhered to human skin. The solvent acrylic adhesive can be attached to the skin for a very long term even under moving cases (at least 5 h in our experiment). Additionally, the PU film is breathable and waterproof, so it can ensure wearing comfortability and proper operation while sweating. We adopted a wearable metabolic system (COSMED, K5, Italy) to record the respiration rate and heart rate, and the ground truth of the BP measurement was the traditional auscultation method. The mounting area and adhesion setup of the sensor is shown in Figure 6a.

A clear baseline can be observed in the original pulse wave, as shown in Figure 6b. After being processed by wavelet transformation, the baseline was successfully extracted, as shown in Figure 6c, where the pulse peaks were also extracted to calculate HR. These two indicators were the same as the output of the wearable metabolic system. The extracted RR (baseline) can be clearly observed in Figure 6d. From the frequency spectrum in Figure 6e, the base frequency (1.2 Hz), and all of its frequency components could be accurately extracted. In Figure 6f, the PWV was estimated by the pulse transition time (PTT) and position distance *D* of the two sensors:

(4) $$PWV=\frac{D}{PTT}$$

With the assistance of least square estimation, the estimation equation of BP can be written as:

(5) BP=4.154×PWV+64

Compared to the results of traditional auscultation methods, the R-square of the proposed estimation equation was 0.7722. For comparison, the R-squares from the estimation equation generated by the commercial available device (non-wearable) [40] distributed in the range of [0.6387, 0.8549]. In this term, the proposed sensor system can obtain the same BP estimation accuracy as the commercial available system.

By using this sensor, vital information for cardiovascular monitoring can be successfully extracted in real time. The PU film can tightly adhere the sensor to human skin with excellent air permeability and robust attachability. In this way, user comfortability is escalated and the sensor position is not prone to change during high-intensity sports activity, which plays an important role in sports, biomedical, and other research.

## 5. Conclusions

In this paper, we introduce a flexible pressure sensor based on porous graphene for continuous cardiovascular status monitoring. The porous graphene was ink-printed, which enabled a fully automatic manufacture procedure for this sensor. The sensor was protected by a PET/EVA-laminated plastic film from severe shear force environments upon human skin. Due to the fine system design, the sensor reached the desired electromechanical performance and excellent sensing ability. To demonstrate its cardiovascular status monitoring ability, we recruited a healthy volunteer and tested his cardiovascular status, including heart rate, respiration rate, and blood pressure. The results indicated that the proposed sensor system can appropriately detect the cardiovascular status of the volunteer, which suggests great application potential in health engineering, precision medicine, and chronic disease rehabilitation. In future work, the disturbance caused by human motion during sports activities will be investigated and tackled.

## Figures and Tables

**Table 1:** State-of-the-art pressure sensors based on graphene.

Sensors	GF	Range	Resolution	Ref.
In-situ foaming PG	53.99 MPa^−1^	1 MPa	<300 Pa	This work
Graphene sheets embedded porous carbon film	130 MPa^−1^	20 kPa	<625 Pa	[33]
Ecoflex/graphene nanoplatelet foams	280 MPa^−1^	10 kPa	-	[31]
Graphene oxide sponge	7.9 MPa^−1^	15 kPa	75 Pa	[32]
Graphene aerogel decorated with piezoelectric nanocrystalline films	6.75 MPa^−1^	100 kPa	-	[34]
Triode-mimicking graphene	710 MPa^−1^	285 k	<15 kPa	[16]

**Table 2:** Parameters for modeling the PG and sealing film in ANSYS.

Material	Density	Elasticity Modulus			Shear Elasticity			Poisson Ratio
		E_x_	E_y_	E_z_	G_xy_	G_xz_	G_yx_	
Unit	g/cm^3^	MPa	MPa	MPa	MPa	MPa	MPa	MPa
PG	2.24	10	10	0.1	4.49	0.05	0.05	0.113
Rubber	1.2	2.14	2.14	2.14	0.72	0.72	0.72	0.48
Plastic	1.38	2280	2280	2280	826	826	826	0.38

## Data Availability

Data sharing not applicable. No new data were created or analyzed in this study. Data sharing is not applicable to this article.
